# PCDE-Sync: A Time Synchronization Mechanism Based on Partial Clustering and the Doppler Effect for Underwater Acoustic Networks

**DOI:** 10.1155/2022/9554396

**Published:** 2022-03-28

**Authors:** Jianping Wang, Jianwei Ma, Yikun Feng, Qigao Feng, Guohong Gao, Yingying Lv

**Affiliations:** ^1^School of Information Engineering, Henan University of Science and Technology, Luoyang 471023, China; ^2^Postdoctoral Research Base, Henan Institute of Science and Technology, Xinxiang 453003, China; ^3^Henley Business School, Whiteknights Campus, University of Reading, Reading, Berkshire, UK

## Abstract

Time synchronization is the basis of coordination and cooperation in underwater acoustic networks. However, because of the propagation delay, node mobility, and Doppler shift, it is impossible to balance the accuracy and energy consumption simply in water. As a promising technology, partial clustering has high convergence and makes breakthroughs in time synchronization. This paper proposes PCDE-Sync, a novel synchronization mechanism with partial clustering and the Doppler effect. Firstly, a clustering method built on the artificial fish swarm algorithm is presented. It models the cluster construction according to fish's preying, swarming, and following behaviors. Secondly, we design a synchronization mechanism to conduct clock correction and compensation by the Doppler effect. Finally, we compare the performance of PCDE-Sync with the most advanced protocols, namely MU-Sync, MM-Sync, and DE-Sync, in terms of the cumulative error after synchronization, the mean square error under different clock skew and that under distinctive node mobility, and energy consumption. The experimental results show that PCDE-Sync makes a trade-off between accuracy and complexity, which does well in solving synchronization issues.

## 1. Introduction

The underwater acoustic network (UAN) comprises a series of nodes randomly deployed in designated water and communicates by acoustic signals [1]. It is a promising network-based exploration system for underwater applications, such as marine data collection, environmental monitoring, submarine resource survey, auxiliary navigation, and tsunami monitoring [2]. In a UAN, multiple nodes run simultaneously for collaborative processing to gain complete and comprehensive data, i.e., data collected by heterogeneous nodes must be consistent in time. Unfortunately, the local time deviates from the reference clock when the node runs, which is inevitable [3]. Synchronization is the basis of coordination and cooperation, which offsets the difference between the local clock and the reference time base [4, 5].

Because of the discrete deployment of underwater nodes, clustering becomes an essential method for time synchronization in UANs. The clustering algorithm is used to build clusters of different regions. Each group elects a cluster head (CH) in a fixed period. Time synchronization is achieved based on intracluster and intercluster communication [6]. Considering energy and performance, it is necessary to design a lightweight and high-precision synchronization algorithm in underwater systems. However, all nodes participate in electing CHs throughout the survival time, which results in the network varying in a constant state. It is apparent that the cost increases significantly [7], and the survival time reduces. The partial or local clustering is founded on the cluster of the given period. It compares the observation value with the weight factor to quantify whether to perform reclustering. If the observation value is within the weight factor, it guarantees the stability of the original cluster as much as possible. Therefore, the reclustering of all nodes is no longer mandatory. The movement of nodes is limited. In other words, the status of nodes is predictable [8] at a specific time. All in all, partial clustering is a promising technique.

The artificial fish swarm algorithm (AFSA) is one of the best optimization methods inspired by fish's social behaviors [9]. It has many advantages, such as good robustness, global searchability, and tolerance of parameter setting. The Doppler effect occurs when waves radiated, reflected, or received by moving objects shift in frequency [10]. In underwater systems, acoustics are employed for communication. The physical properties of underwater acoustic waves include slow propagation speed and frequency-selective signal-to-heat conversion, causing only low-frequency signals to propagate over long distances. Therefore, the underwater acoustic channel is unreliable with small capacity and long delays [11]. The low propagation speed (about 1500 m/s) causes a significant Doppler shift. With this motivation, we try to model underwater nodes as random fish swarms, use AFSA to achieve partial clustering, and build a novel time synchronization mechanism by the Doppler effect, namely PCDE-Sync. The main contributions are summarized as follows:

1. We model the UAN as multiple fish swarms and propose a partial clustering algorithm based on AFSA.
2. We design a CH selection mechanism according to the preying, swarming, and following behaviors of the fish.
3. We present a time compensation and correction method based on the Doppler effect.

To the best of our knowledge, PCDE-Sync is the first work for time synchronization that combines partial clustering with the Doppler effect in UANs. Based on this work, the bottleneck of the UAN is solved. In addition, the distance from a CH to a data node (DN) is less than that to the surface buoy. At the same time, significant energy saving is achieved. Finally, it maintains the stability of the UAN, which is another necessary guarantee. In short, a trade-off between accuracy and complexity is obtained in PCDE-Sync for solving time synchronization issues.

The rest of the paper is arranged as follows: section 2 describes the related work.  Section 3 introduces the partial clustering algorithm.  Section 4 presents the time synchronization mechanism.  Section 5 discusses the simulation experiments. Finally, Section 6 concludes the paper.

## 2. Related Work

### 2.1. The Dynamic Synchronization Mechanisms

The nodes are usually mobile and affected by ocean currents in UANs. For dynamic synchronization algorithms, they generally simulate node mobility by a given time base and use the filter tracking methods to correct and compensate the clock deviations [12–14].

Ni et al. [15] proposed K-Sync, a pairwise synchronization algorithm based on the Kalman filter. The simulation shows that K-Sync is robust to various underwater motion scenes. Liu et al. [16] presented a time synchronization method based on clock skew tracking. The simulation results show that the accumulative root mean square error (RMSE) reduces. Wei et al. [17] introduced a clock compensation algorithm based on the BP network. The synchronization compensation algorithm is established for error backpropagation. Simulation experiments show that accuracy increases, and the mean square error reduces. Wang et al. [18] proposed a joint estimation scheme for fine timing, Doppler scaling factor, and carrier frequency offset based on pilot symbols. The method is performed in a block-by-block fashion. Experimental results show decent detection performance for data with relative transceiver motion.

Syed et al. [19] introduced a time synchronization protocol for a high-latency (TSHL) environment. The simulation results demonstrate that it achieves twice the accuracy with minimum energy consumption at 500 meters. Huang et al. [20] proposed an improved synchronization algorithm that combines the mobility of unknown nodes to reduce the clock deviation. The experiment is practical and feasible. Wang et al. [21] studied a dynamic synchronization algorithm with the current model. Experiments reveal the reliability and practicability of the algorithm. Lin et al. [22] considered a motion-based synchronization method (MM-Sync) for underwater sensor networks. Experimental results showed that it reduces energy consumption and has higher accuracy. Zhou et al. [23] introduced an adaptive synchronization algorithm (APE-Sync) in underwater sensor networks. The simulation showed that it was a high-precision, low-cost synchronization scheme. Liu et al. [24] conducted a synchronization scheme for mobile UANs. The results show that it achieves higher accuracy with lower overhead. Sumin et al. [25] presented TSUC, a time synchronization algorithm based on the beacon message interval and the skew estimation of root mean square (RMS) in UANs. Experiments show that it confirms accuracy comparable to TSHL but more straightforward skew estimation. Chen et al. [26] presented a synchronization protocol for underwater acoustic networks. The simulation proves that it achieves better performance than TSHL. Liu et al. [27] established a receiver-only synchronization (ROS) model and designed a method to estimate the clock offset and the deviation of active nodes and silent ones. Simulation shows that it has high accuracy and is more robust.

The studies mentioned above reveal the accuracy and reliability of dynamic synchronization methods in given scenarios. Nevertheless, it is still a significant challenge because of the high propagation delay, the estimation model, and the synchronization framework. On the one hand, the cumulative error of these methods is serious. As nodes increase, the accuracy will be significantly reduced [28]. On the other hand, iterative operations are performed and convergence is poor. In addition, they rely heavily on the reference clock and assume that the time base has good performance. However, they often use the crystal oscillator as the clock source. The frequency of oscillators is easily affected by electronic noise and the underwater environment and is prone to clock drift [29].

### 2.2. Cluster-Based Synchronization Methods

Clustering algorithms are the basis for hierarchical network management. It improves scalability and dramatically promotes the coverage and connectivity of underwater systems.

Shivaraman et al. [30] proposed C-Sync, a clustering-based time synchronization protocol that provides resilience against faults with energy-efficient communication. Experiment results show that C-Sync detects and isolates faults to a cluster and recovers quickly. Alsolami et al. [31] designed a cluster-based synchronization method for self-synchronized drone networks. The experiment shows that the proposed algorithm has high prediction accuracy. Omeke et al. [32] presented a protocol called distance and energy-constrained k-means clustering scheme (DEKCS) for clustering, cluster-head selection, and data retrieval to prolong the survival time of underwater networks. Evaluations show that DEKCS outperforms the low-energy adaptive clustering hierarchy (LEACH) protocol.

Chirdchoo et al. [33] suggested a cluster-based synchronization algorithm for mobile UANs (Mu-sync that considers long-term and time-varying propagation delays and estimates clock deviations through linear regression. The simulation results show that the delay estimation determines the accuracy to a large extent. Wu et al. [34] recommended a cluster-based synchronization algorithm. The simulation results show that the combination of cluster topology reduces network flow and improves convergence. Wang et al. [35] proposed a synchronization scheme that includes three algorithms, i.e., threshold-based intracluster time synchronization (TITS), forwarding-based intercluster time synchronization (FITS), and one-way intracluster time synchronization (OITS). The simulation results show that the synchronization scheme reduces the flow rate and improves the convergence. Based on clustering technology, Jia et al. [36] constructed a chained time synchronization protocol for the sensor network (C-TPSN). Experimental results show that the performance of C-TPSN is better than the timing-sync protocol for sensor network (TPSN). Anand et al. [37] studied an underwater synchronization protocol that uses an autoregressive model to transmit data. The simulation results show that it achieves network-wide synchronization when compared with TSHL. Yuan et al. [38] simulated the three-message synchronization protocol in the UAN. The results show that the protocol performs better overall. Kong et al. [39] proposed a dual-channel synchronization mechanism (DCH-sync) that achieves synchronization through a mobile beacon and dual CHs. Xu et al. [40] proposed a cluster-based secure synchronization (CLUSS) method. Simulation results show that it decreases errors and reduces synchronization messages.

Although these studies demonstrate the benefits of cluster-based mechanisms, there are still shortcomings. Firstly, they are most concerned with the CH electing and the network stability. Secondly, frequent communication will cause bottlenecks in centralizing CHs. However, there is no ideal time-compensation solution with sparse nodes in UANs. Some dynamic algorithms have no systematic trigger and reclustering methods. Taking into account the cost and stability, the partial clustering algorithm becomes a strategic choice. Unfortunately, few studies have focused on it [41].

### 2.3. Time Synchronization Based on the Doppler Effect

Because of the low propagation of acoustic signals, even a slight node movement causes a significant Doppler shift in UANs. Therefore, various synchronization methods are recommended based on the Doppler effect.

Yao et al. [42] proposed CD-Sync, a time synchronization algorithm based on the clustering and Doppler velocity measurement in the water. Experimental results show that CD-Sync shortens the distance among nodes and accelerates convergence speed while effectively improving synchronization accuracy. Yang et al. [43] introduced a non-data-aided Doppler estimation method for underwater systems with M-ary spread. The simulation results show that the proposed algorithm is better than the sliding correlation estimation method with average Doppler compensation. Kim et al. [44] offered a sequencing technique for the joint estimation of accurate timing and the cell ID in underwater acoustic systems with a high Doppler effect. A generalized Zadoff-Chu sequence is decomposed into multiple subsequences for reducing the Doppler effect. Lu et al. [45] presented a joint Doppler estimation and compensation method based on spectrum zooming and correction. The simulation results show that the proposed mechanism improves the estimation accuracy and increases the cross-correlation processing gain.

Lu et al. [46] introduced a Doppler shift-based synchronization protocol (D-Sync) for mobile UANs. The simulation results show that D-Sync is superior to existing methods for accuracy and energy consumption. Sidorkina et al. [47] presented a synchronization algorithm using the fractional Fourier transform (FrFT) in UANs. The simulation shows the synchronization possibility under different Doppler factors. Do et al. [48] proposed a synchronization method in OFDM-based underwater systems. Experiments show that the performance of the proposed method is better than the Schmidls algorithm. Kebkal et al. [49] design a synchronization method during payload exchange in UANs. The results show that data exchange is invoked as a comfortable way to the time synchronization. Wang et al. [50] model the hyperbolic frequency modulation (HFM) signal as channel probes for the Doppler estimation. The results show that the synchronization mechanism has better accuracy. Trubuil et al. [51] propose an estimation technique with a dual-training sequence of the Doppler shift. Experiments show that the method is effective in acoustic communication. Balakhonov et al. [52] present a Doppler estimation algorithm for underwater OFDM signals. It considers the multipath propagation of signals with discrete Doppler scales. Li et al. [53] study an estimation algorithm that combines synchronization and the Doppler scale in underwater acoustic communication with the Zadoff-Chu sequences. The experiment proves the effectiveness and robustness of the method. Zhou et al. [54] established a Doppler-enhanced synchronization (DE-Sync) scheme for mobile underwater sensor networks. The results show that the accuracy and energy efficiency are more significant than the existing synchronization protocol. Pallares et al. [55] designed a synchronization algorithm that considers the Doppler scale estimation in UANs. Liu et al. [56] proposed a cross-layer synchronization scheme for mobile UANs, which uses the Doppler effect and a Kalman filter to estimate the propagation delay. The simulation results show that it obtains a higher accuracy with minimal overhead. At present, we carry out related research on time synchronization based on the Doppler effect in UANs. We construct a synchronization mechanism based on the Doppler effect under the condition of relative motion of a single node and the buoy. Through data analysis, we draw a conclusion that the depth of the nodes has a greater effect on time synchronization [57].

The above studies consider the Doppler estimation and compensation in time synchronization. They verify the effectiveness in given scenarios from different aspects, such as signal quality, capacity, and attenuation. However, they are complex and rely on advanced hardware infrastructures. Also, most of them ignore timeliness. Finally, it is worth mentioning that there are problems in designing underwater nodes with limited energy.

## 3. A Partial Clustering Mechanism of the Artificial Fish Swarm Algorithm

### 3.1. A UAN Scenario for Time Synchronization

**Figure 1:**
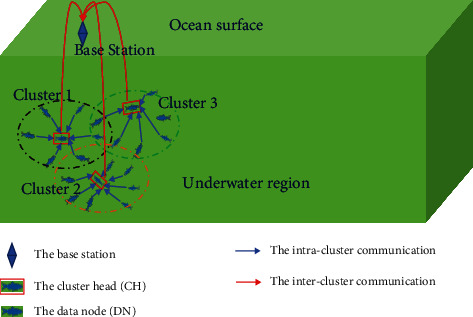
A UAN scenario for time synchronization.

Suppose a UAN is deployed in a fixed underwater area and each node is equipped with an anchor chain and an anchor. When the node sinks into the water, the anchor chain scales to a certain length according to the depth gauge. A depth sensor and a pressure sensor are settled in the node. As the node is battery-powered, it fails if the energy is exhausted. When all nodes are in place, the surface buoy or the base station (BS) broadcasts a cluster-initial message and triggers the partial clustering mechanism. After a period, the UAN is divided into independent clusters, and each one elects a CH. After that, the time synchronization starts. The communication scene is shown in Figure 1.

**Figure 2:**
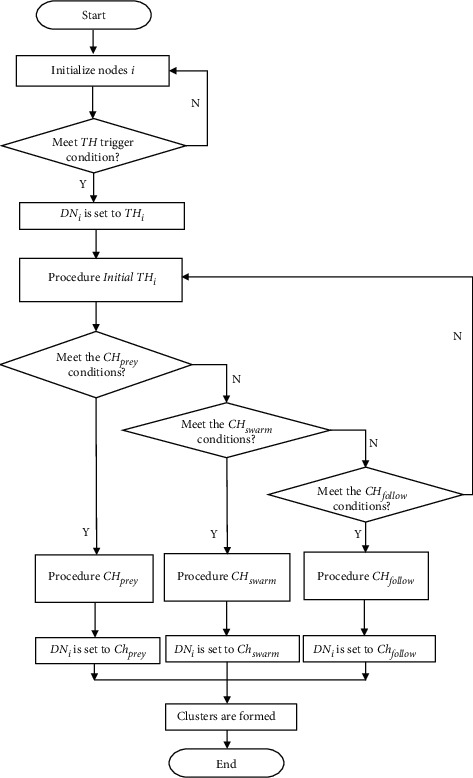
The flow of the partial clustering mechanism.

Selecting a temporary head (TH) is the first step of the partial clustering algorithm. Next, the electing of CHs is achieved by modeling the preying, swarming, and following behavior of the fish. Messages designed for the clustering and synchronization algorithm are listed in Table 1. The flow of the algorithm is shown in Figure 2.

### 3.2. Cluster Initialization

There are three roles for a node in the clustering algorithm, namely DN, TH, and CH. DN is the default role. The BS broadcasts a cluster-initial message during the initialization process. The DN receives the message and triggers the clustering algorithm within the communication range.

The threshold of a TH (TH_Temp_CH_) is included in the cluster-initial message, which is settled on BS for electing TH, as shown in the following equation:

(1) $$\begin{array}{c} {\text{TH}}_{\text{Temp\_CH}}\left(n\right)=\left\{\begin{array}{cc} \frac{P}{1-P\times \left(T_{\text{Round}}\mathrm{mod}1/P\right)}, & n\in S,\\ 0, & \text{others,} \end{array}\right. \end{array}$$

where *P* represents the CH ratio, *T*_Round_ represents the execution round, and *S* represents the set of DNs.

The node that is selected as TH or CH will be signed in each round. In the next turn, it will never act on these roles. *τ*_Temp_CH_ is applied to all nodes. The DN that receives the cluster-initial message will generate a random number *r* between 0 and 1. Then, it extracts *τ*_Temp_CH_ from the message and compares it with *r*. If *r* > *τ*_Temp_CH_, then the node preserves the DN role and waits to join a relevant cluster. Otherwise, it immediately changes its role to TH and broadcasts a temporary-head-request message. The communication capability (CC), node ID, and timestamp are encapsulated in this message, where CC is measured by the residual energy, CPU utilization, and storage occupancy rate, as shown in the following equation:

(2) CCi=W1×EResi+W2×ECPUi+W3×EStoragei,

where *E*_RES_(*i*) is the expectation of residual energy, *E*_CPU_(*i*) is CPU utilization, and *E*_*Storage*_(*i*) is the occupancy ratio of storage. *W*_1_, *W*_2_, and *W*_3_ are weight factors.

Followed by this, TH broadcasts a compete–CH–request message. The DN centered on TH will receive this message, extract its CC according to (2), and respond with a compete–CH–response message. The CC and node ID are encapsulated in this message. The destination of the message is to the specified TH.

A DN may be in the cross-region of multiple clusters. At this time, numerous compete–CH–request messages will be received. The node will select the nearest TH according to the CC of the received compete–CH–request messages. The TH with a minor ID is preferred if these messages have the same CC. Then, TH will send a compete–CH–response message. DN will sign this node as its TH, and other nodes will be ignored.

If nodes are sparse in UAN, there may be no DN in the cluster. In this case, the compete–CH–request message sent by TH will not receive any response. Therefore, a timer starts when TH sends the compete–CH–request message. If no message is received within the given period, TH will be marked as an orphan node, and it will immediately change its role to DN and add a lock flag. It means that the node no longer participates in CH selection and only joins other clusters by receiving compete–CH–request messages. The pseudocode of the cluster initialization is shown in Algorithm 1.

### 3.3. CH Selection Based on Preying Behavior

Studies have shown that fish can efficiently identify locations and move synchronously through mutual learning. As an optimization method, AFSA is inspired and abstracted by fish behavior. It is shown that AFSA has parallel computing capabilities, high convergence, flexibility, fault tolerance, and can quickly obtain feasible solutions.

In the initialization phase, multiple clusters are formed, and each cluster is a 3D area with a TH as its center. DN is signed with the ID of TH in the cluster. In a short period, the node status is roughly the same, i.e., there is almost no difference in relative motion. Therefore, the cluster is assumed to be stable for a certain period.

Afterward, it models and simulates the behaviors of fish for electing CHs. The location of the TH is represented as a vector, where $p_{i}\left\{\begin{array}{c} x_{i}\left(i=1,2,\ldots ,n\right)\\ y_{i}\left(i=1,2,\ldots ,n\right)\\ z_{i}\left(i=1,2,\ldots ,n\right) \end{array}\right\}$ is the location that needs to be found. The CH selection based on preying behavior is quantified as the residual energy of THs and their distances to BSs. At first, each CH assigns a location randomly. Assuming that the current location of the TH is *P*_*i*_(*t*), the following location *P*_*rand*_ is selected within its sensing range, as shown in the following equation:

(3) $$\begin{array}{c} P_{rand}=P_{i}\left(t\right)+Ran\left(\;\right)\times Visual, \end{array}$$

where *V*isual is the visibility range.

If the residual energy of the nearest DN is greater than TH, the node is selected as a preying-based CH, namely CH_prey_. At this time, the progressive location of TH is shown in (4).

(4) $$\begin{array}{c} P_{i}\left(t+1\right)=P_{i}+\mathrm{Rand}\left(\;\right)\times CH_{\mathrm{Step}}\times \frac{P_{j}-P_{i}}{\left\|P_{j}-P_{i}\right\|}, \end{array}$$

where *CH*_*Step*_ means the forwarding step, and ‖*P*_*j*_ − *P*_*i*_‖ is the Euclidean distance between *P*_*j*_ and *P*_*i*_.

Otherwise, the TH continues to look for another random location, as shown in the following equation:

(5) $$\begin{array}{c} P_{i}\left(t+1\right)=P_{i}\left(t\right)+Ran\left(\;\right)\times Step. \end{array}$$

If the distance from *P*_*i*_(*t*+1) to BS is less than that to TH and the residual energy is greater than TH, then it is elected as CH_prey_. If no DN matches the condition, the TH randomly moves to the next step and marks itself as CH_prey_. After selecting CH_prey_, it broadcasts a statistics-node-request message, and the relevant DNs reply to it with a statistics-node-response message and mark the CH_prey_. Finally, CH_prey_ registers the cluster to a designated BS. If no node corresponds to the condition, it returns to the process of cluster initialization. The pseudocode of CH selection based on preying behavior is shown in Algorithm 2.

### 3.4. CH Selection based on Swarming Behavior

Multiple CHs are elected based on preying behavior in the previous stage. A CH is registered on BS in each iteration to update its status to obtain the network's global view. In a cluster, a DN sends data to a specified CH_prey_. CH_prey_ needs to receive, aggregate, verify, process, and transmit data to a corresponding BS. Therefore, the energy consumption of CH_prey_ is rapid. Once the energy is exhausted, CH_prey_ fails, and the communication will be interrupted. A threshold *E*_*τ*_ is settled on CH_prey_ to balance energy consumption. If the residual energy of CH_prey_ is lower than *E*_*τ*_, i.e., *E*_*CH*_prey__ < *E*_*τ*_, it will select another CH and name it as CH_swarm_.

In the cluster, CH_prey_ broadcasts a node-location-request message. DNs respond to a node-location-response message. Therefore, it is easy to gain the center location *P*_*C*_, as shown in the following equation:

(6) $$\begin{array}{c} P_{C}=\left\{\begin{array}{c} X_{C}=\frac{1}{n}\sum_{i=1}^{n}x_{i}\\ Y_{C}=\frac{1}{n}\sum_{i=1}^{n}y_{i}\\ Z_{C}=\frac{1}{n}\sum_{i=1}^{n}z_{i} \end{array}\right.. \end{array}$$

The average residual energy *E*_*average*_ of DNs is shown in the following equation:

(7) $$\begin{array}{c} E_{\text{average}}=\frac{1}{n}\sum_{i=1}^{n}Node_{i}. \end{array}$$

At this time, *CH*_*prey*_ chooses the DN closest to *P*_*C*_, the residual energy greater than *E*_*average*_ as the next CH, namely *CH*_*swarm*_, and inferences the improved location as shown in the following equation:

(8) $$\begin{array}{c} P_{i}\left(t+1\right)=P_{i}\left(t\right)+Ran\left(\;\right)\times Step\times \frac{P_{C}-P_{i}\left(t\right)}{\left\|P_{C}-P_{i}\right\|}. \end{array}$$

After selecting the *CH*_*swarm*_, it broadcasts a statistics-node-request message, and the relevant DNs respond to it with a statistics-node-response message and mark the *CH*_*swarm*_. Finally, it registers the cluster to a designated BS. If no node corresponds to the condition, it returns to the process in the previous stage. The pseudocode of the CH selection based on swarming behavior is shown in Algorithm 3.

### 3.5. CH Selection Based on Following Behavior

After selecting CHs by the swarming behavior, CH_swarm_ is located in the center of a cluster. However, the topology may change in the next iteration. Therefore, CH_swarm_ may no longer be the center of the cluster. CH_swarm_ broadcasts a residual-energy-request message within a given period to detect cluster changes. DNs in the cluster respond to a residual-energy-response message. CH_swarm_ unpacks the message that it receives. The threshold for selecting CHs as *T*_CH_follow__ based on the following behavior is given, and assume that the number of residual-energy-response messages received by the CH_swarm_ is *χ*. If *χ* < *T*_CH_follow__, it means that the cluster has changed a lot. In this case, it is necessary to find the next optimal CH, i.e., CH_follow_, according to the following behavior. The residual energy, the node ID, and the location information are encapsulated in the residual-energy-response message.

The *CH*_swarm_, firstly, finds a DN with enormous residual energy by processing the residual-energy-response message it receives. Then, it takes this node as the center and calculates other nodes within its communication radius.

Suppose that the number of DNs centered on CH is *N*(*i*)_*count*_. *T*_*crowd*_ represents the congestion factor. If *N*(*i*)_*count*_ < *T*_*crowd*_ and the location is closest to *CH*_swarm_, the node will be selected as *CH*_*follow*_, and the location will be updated, as shown in the following equation:

(9) $$\begin{array}{c} P_{i}\left(t+1\right)=P_{i}\left(\text{t}\right)+Ran\left(\;\right)\times Step\times \frac{P_{M\text{ax}}-P_{i}\left(\text{t}\right)}{\left\|P_{M\text{ax}}-P_{i}\right\|}, \end{array}$$

where *P*_*M*ax_ represents location, the residual energy of which is the largest.

After selecting *CH*_*follow*_, it broadcasts a statistics-node-request message, and the relevant DNs respond to it with a statistics-node-response message and mark *CH*_*follow*_. Finally, the *CH*_*follow*_ registers the cluster to a designated BS. If no node corresponds to the condition, it returns to the process in the previous stage. The pseudocode of the CH selection based on the following behavior is shown in Algorithm 4.

## 4. A Time Synchronization Method Based on the Doppler Effect

### 4.1. The Procedure of Synchronization Mechanism

**Figure 3:**
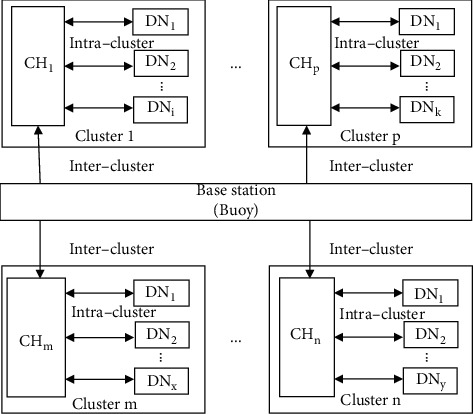
The diagram of the synchronization mechanism.

After a cluster is established, CH transmits the statistics-node-request message to the DNs of its domain. The ID of CH is encapsulated in this message. Since DNs are registered with CH when the cluster is formed, they alone would receive this message. Then, the corresponding DNs reply to the statistics-node-response message, which records the statistics of DNs. CH aggregates statistics-node-response messages and sends a cluster-statistics message to BS. Followed by this, it establishes the clock correction and compensation based on the Doppler effect. The procedure mainly includes two phases, i.e., intercluster synchronization and intracluster synchronization. A schematic diagram of the synchronization mechanism is shown in Figure 3.

#### 4.1.1. Intercluster Synchronization Phase

The intercluster synchronization is performed to gain the reference clock base. Firstly, BS broadcasts an intersync-trig message to trigger the synchronization process, and the local clock is encapsulated in this message. Multiple CHs receive intersync-trig messages and send intersync-req messages to BS. After receiving the intersync-req message, BS responds to an intersync-ack message. The intercluster synchronization is achieved by the interaction of intersync-req messages and intersync-ack messages. In this process, when the CH receives the intersync-trigger message, it, firstly, records the signal transmission time and its local clock and estimates the propagation delay between it and the BS.

#### 4.1.2. Intracluster Synchronization Phase

CH is established in intercluster synchronization. Because of the network changes, CH needs to communicate with BS within a given period to gain the latest clock. DNs are generally in sleep mode for energy saving. When a sensing event occurs, DNs will first send an intrasync-req message, including its ID and transmission time. If the CH receives these messages, it responds to an intrasync-ack message that contains the reply time. DNs estimate the propagation delay to correct the clock skew in the intercluster synchronization phase.

### 4.2. Time Correction and Compensation Based on the Doppler Effect

If there is motion between the wave source and the observer, the Doppler effect will occur. In UANs, even tiny motions will cause a noticeable Doppler effect. Nevertheless, it provides a novel way for time synchronization. Let *F*_*S*_ be the frequency of the wave source, *λ* be the wavelength, *V*_*w*_ be the wave velocity, *V*_*o*_ be the velocity of the observer, and *V*_*s*_ be the velocity of the wave source. Assume that *V*_*w*_, *V*_*o*_, and *V*_*s*_ have directivity. In the Doppler effect, there are three situations in which the observer is stationary, the wave source is static, and the wave source and the observer move relative to each other.

In the first case, when the wave source is close to a stationary observer, the wave velocity is *V*_*w*_ − *V*_*s*_ and the wavelength is *λ*′=*V*_*w*_ − *V*_*s*_/*F*_*s*_. However, the observer recognizes that the wave velocity is *V*_*w*_ and the wavelength is *λ*′. Therefore, the observer gets the frequency *F*=*V*_*w*_/*λ*′, as follows:

(10) $$\begin{array}{c} F=F_{s}\times \frac{V_{w}}{V_{w}-V_{s}}. \end{array}$$

In the second case, the observer treats the velocity as *V*_*w*_ − *V*_*o*_ and the wavelength as *λ*=*V*_*w*_/*F*_*s*_. Therefore, the frequency is *F*=*V*_*w*_ − *V*_*o*_/*λ*, as shown in the following equation:

(11) $$\begin{array}{c} F=F_{s}\times \frac{V_{w}-V_{o}}{V_{w}}. \end{array}$$

In the third case, the observer and the wave source are close to each other. Therefore, the frequency is as follows:

(12) $$\begin{array}{c} F=F_{s}\times \frac{V_{w}-V_{o}}{V_{w}-V_{s}}. \end{array}$$

**Figure 4:**
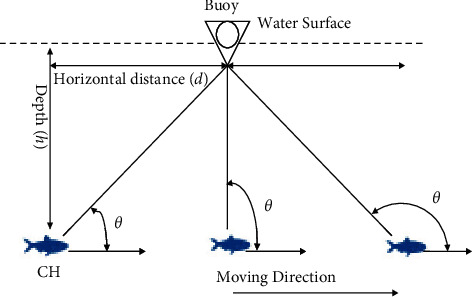
The relation between a CH and the buoy.

Assume that the horizontal distance between a CH and the buoy is *d* in the beginning, and the CH is at depth *h*. Let *V*_*o*_ represent the moving velocity. *F*_*s*_ is affected by the Doppler effect. The horizontal angle *θ* will affect the frequency *F*(*t*) if the wavelength remains the same, as shown in Figure 4.

Therefore, the frequency *F*(*t*) can be derived, as shown in the following rquation:

(13) $$\begin{array}{c} F\left(t\right)=F_{s}\times \frac{V_{w}+V_{o}\times \cos\theta \left(t\right)}{V_{w}}, \end{array}$$

where *F*(*t*) is related to cos*θ*(*t*). If CH is infinitely far from the buoy, then *θ*=0 and cos*θ*(*t*)=1. Therefore, the maximum frequency *F*_max_ is shown in the following equation:

(14) $$\begin{array}{c} F_{\max}=F_{s}\times \frac{V_{w}+V_{0}}{V_{w}}. \end{array}$$

If CH is orthogonal to the buoy, the intersection angle is *θ*=*π*/2 and cos*θ*(*t*)=0. The frequency in this case is *F*_*s*_. If CH is located infinitely behind the buoy, then cos*θ*(*t*)=−1. Therefore, the minimum frequency *F*_min_ is shown in the following equation:

(15) $$\begin{array}{c} F_{\min}=F_{s}\times \frac{V_{w}-V_{o}}{V_{w}}. \end{array}$$

*T*_0_ is given as the detection time when the frequency is close to *F*_*s*_. Therefore, CH reaches just below the buoy at *T*_0_, and *F*(*t*) is shown in the following equation:

(16) $$\begin{array}{c} F\left(t\right)=F_{s}\times \frac{V_{w}-V_{o}\times V_{0}\times T_{t}/\sqrt{{\left(V_{0}\times T\right)}^{2}+h^{2}}}{V_{w}}. \end{array}$$

The difference between *F*(*t*) and *F*_min_ is below the threshold *F*_*σ*_. It must comply with the following:

(17) $$\begin{array}{c} F\left(t\right)\leq F_{\min}+F_{\sigma }. \end{array}$$

Therefore, it solves the range of *T*_*t*_ from (15), (16), and (17). The result is as follows:

(18) $$\begin{array}{c} T_{t}\geq \frac{1}{V_{0}}\times \sqrt{\frac{{\left(1-V_{w}\times F_{\sigma }/V_{0}\times F_{s}\right)}^{2}\times h^{2}}{1-{\left(1-V_{w}\times F_{\sigma }/V_{0}\times F_{s}\right)}^{2}}}. \end{array}$$

By finding the minimum time *T*_*t*_, it solves *F*_min_, which is closest to ${\bar{F}}_{\min}$. Assuming that *n* dataset has been settled, then ${\bar{F}}_{\min}$ is defined in (19).

(19) $$\begin{array}{c} {\bar{F}}_{\min}=\frac{1}{n}\sum_{i=1}^{n}F_{i}\left(t\right). \end{array}$$

After that, it estimates $\tilde{V_{o}}$ as shown in (20).

(20) $$\begin{array}{c} \tilde{V_{o}}=V_{w}\times \left(1-\frac{{\bar{F}}_{\min}}{F_{s}}\right). \end{array}$$

When *t*=0, CH is located at distance *d* in front of the buoy. If *t*=*T*_0_, CH just passes the buoy and moves at the velocity of *V*_0_ during this period. Suppose that $\tilde{V_{o}}$ is the closest to *V*_0_, it is clear that $\tilde{d}$ is the closest to *d*, as shown in the following:

(21) $$\begin{array}{c} \tilde{d}={\tilde{V}}_{0}\times T_{0}. \end{array}$$

By substituting ${\tilde{V}}_{0}$ in (13), we get Ζ, which calculates the detection point, as shown in the following equation:

(22) $$\begin{array}{c} Ζ=\frac{V_{w}\times \left(\tilde{F}\left(t\right)-F_{s}\right)}{{\tilde{V}}_{0}\times F_{s}}. \end{array}$$

Next, we define Ζ as cos*θ*′(*t*), and the intersection angle is easily derived. As there will be some detection errors when Ζ exceeds the interval [−1,1], cos*θ*′(*t*) is defined as follows:

(23) $$\begin{array}{c} \cos\theta^{\prime }\left(t\right)=\left\{\begin{array}{cc} 1 & Ζ>1\\ Ζ & -1\leq Ζ\leq 1\;\\ -1 & Ζ<-1 \end{array}\right.. \end{array}$$

According to the trigonometric function, it transforms cos*θ*′(*t*) into $\tan\tilde{\theta }\left(t\right)$, as shown in the following equation:

(24) $$\begin{array}{c} \tan\tilde{\theta }\left(t\right)=\frac{\sqrt{1-\cos^{2}\theta^{\prime }\left(t\right)}}{\cos\theta^{\prime }\left(t\right)}. \end{array}$$

The relationship between distance *d* and depth *h* is expressed as follows:

(25) $$\begin{array}{c} \tan\tilde{\theta }\left(t\right)=\frac{h}{\tilde{d}-\tilde{V_{0}}\times t}. \end{array}$$

By substituting (21) in (25), $\tan\tilde{\theta }\left(t\right)$ is expressed as follows:

(26) $$\begin{array}{c} \tan\tilde{\theta }\left(t\right)=\frac{h}{\tilde{V_{0}}\times T_{0}-\tilde{V_{0}}\times t}\\ =\frac{h}{\tilde{V_{0}}}\times \frac{1}{T_{0}-t}. \end{array}$$

Assume that *x*=1/*T*_0_ − *t*. Then, (26) can be rewritten as follows:

(27) $$\begin{array}{c} \tan\tilde{\theta }\left(x\right)=\frac{h}{{\tilde{V}}_{0}}\times x. \end{array}$$

It is a linear relationship between $\tan\tilde{\theta }\left(x\right)$ and *x* in (27). The coefficient $h/\tilde{V_{0}}$ is solved by removing the meaningless point *t*=*T*_0_ and substituting $\tan\tilde{\theta }\left(x\right)$ in (27).

If CH receives the synchronization message, it, firstly, records the start time *T*_*b*_ when BS transmits signals. At the same time, CH records its own time *T*_*s*_. Then, it solves *V*_0_, *d*, and *h*. Therefore, the propagation delay *T*_*d*_ can be obtained as shown in the following equation:

(28) $$\begin{array}{c} T_{d}=\frac{\sqrt{d^{2}+h^{2}}}{V_{w}}. \end{array}$$

As CH is constantly in motion, the propagation delay will change. When *T*_*d*_ is solved, the clock offset between BS and CH is counted as follows:

(29) $$\begin{array}{c} \Delta T=T_{b}-T_{s}+T_{d}. \end{array}$$

Finally, it adds Δ*T* to the system time and completes the synchronization process.

## 5. Simulation Experiments

In this paper, we construct a UAN scenario in WOSS-NS3, in which 100 nodes are randomly deployed. We compare MU-Sync, MM-Sync, and DE-Sync with PCDE-Sync in the experiment because these algorithms coexist with PCDE-Sync. For MU-Sync, it is the first cluster-based synchronization algorithm. For MM-Sync, it is a representative synchronization method. DE-Sync is a Doppler-enhanced synchronization scheme. The main experimental parameters are shown in Table 2.

### 5.1. Cumulative Error after Synchronization

In a UAN, underwater nodes obtain synchronized clocks from reference time bases. UAN based on a hierarchical structure usually uses clustering and other methods to build local synchronization, and then gradually expands to the whole system. The cumulative error is usually measured by the time difference between the local node and the reference clock. For PCDE-Sync, each node maintains a physical clock and a global logical clock. The cumulative error of PCDE-Sync mainly occurs when CH requests to establish synchronous communication with the surface buoy, the exchange of synchronization among CHs, and the synchronization between DNs and CHs based on intracluster communication. The cumulative error is shown in the following equation:

(30) $$\begin{array}{c} C_{i}^{*}=C_{i}^{\text{local}}-C_{i}^{\text{ref}}-C_{i}^{CH}. \end{array}$$

where *C*_*i*_^*local*^ represents the local clock of a DN, *C*_*i*_^*ref*^ means the reference clock, and *C*_*i*_^*CH*^ is the local clock of the corresponding CH.

Therefore, for an underwater sensor network with *m* nodes, the average clock skew and offset can be expressed as follows:

(31) $$\begin{array}{c} \alpha_{S\text{kew}}=\frac{\sum_{i=1}^{m}\left(C_{i}^{*}-\bar{C_{i}}\right)\left(C_{i}^{ref}-\bar{C^{ref}}\right)}{\sum_{i=1}^{m}{\left(C_{i}^{*}-\bar{C_{i}}\right)}^{2}},\\ \beta_{offset}=\bar{C^{ref}}-\alpha_{Skew}\times \bar{C_{i}}, \end{array}$$

where $\bar{C_{i}}$ is the average value of the local time and $\bar{C^{ref}}$ is the average value of the reference clock.

**Figure 5:**
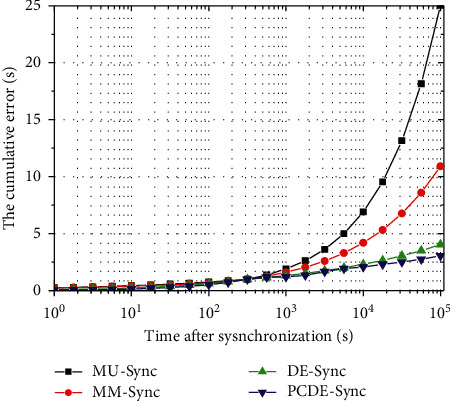
Cumulative errors after synchronization.

Figure 5 shows the cumulative error of the four algorithms after synchronization, which appears with an increasing trend. When the time is before 10^3^, the cumulative error of the four algorithms is approximately the same. When the time is after 10^4^, the cumulative error of MM-Sync and MU-Sync varies quickly. However, those of DE-Sync and PCDE-Sync are flatter. Overall, the cumulative error of PCDE-Sync is about 4.121% smaller than that of MU-Sync, MM-Sync (8.79%), and DE-Sync (1.45%). When the time is after 106, the cumulative error of PCDE-Sync is reduced by 28.3% compared to MU-Sync, MM-Sync (16.1%), and DE-Sync (5.19%).

MU-Sync uses a half-round time to determine the propagation delay, and the synchronization error is the largest. For MM-Sync, it uses the Doppler factor to estimate the relative motion, and the synchronization error is lower than that of MU-Sync. The linear frequency modulation (LFM) and the OFDM symbols are used as the preamble for the initial Doppler scale estimation in DE-Sync. For PCDE-Sync, the partial clustering mechanism is implemented in ASFA. Thus, DNs only exchange with the elected CH, significantly reducing the communication range. In addition, the distances between CHs and BSs are quantified by the Doppler effect, which is beneficial for correcting synchronization errors. Therefore, the cumulative error of PCDE-Sync is the lowest.

### 5.2. MSE Comparison under Different Clock Skew

**Figure 6:**
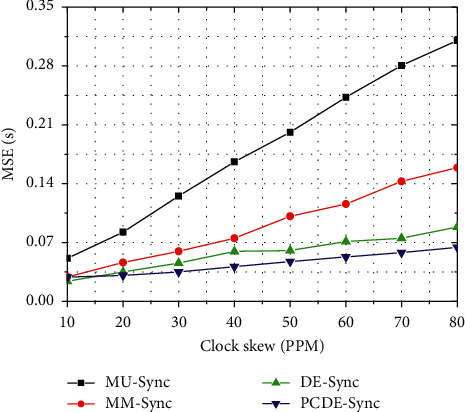
MSE vs. Clock skew.

After the clustering is finished, CH processes the difference between the local clock and the time base. The initial frequency deviation varies from 10 PPM (parts per million) to 80 PPM in this experiment. We see that the MSE of the four algorithms has a rising trend with the increase in frequency skew, as shown in Figure 6.

Within the skew range, the MSE change of MU-Sync is 35.30%, that of MM-Sync is 27.32%, that of DE-Sync is 13.38%, and that of PCDE-Sync is 8.76%. Among the four algorithms, MU-Sync has the largest MSE, and PCDE-Sync has the smallest one. For MM-Sync, the frequency offset is reduced by the linear fitting. For MU-Sync, it estimates the clock deviation by performing linear regression.

Consequently, the clock deviation is lower than that of MM-Sync. For DE-Sync, it estimates the clock offset and offset by substituting the Doppler scale factor into the linear regression. For PCDE-Sync, it forms partial clustering and realizes the clock offset correction and compensation through the Doppler effect in a relatively small region. Therefore, the accuracy is higher than that of DE-Sync.

### 5.3. MSE Comparison under Differential Node Mobility

**Figure 7:**
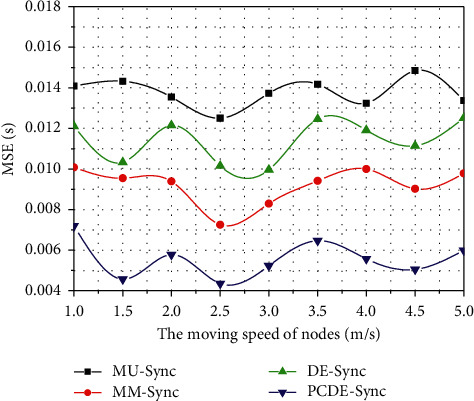
MSE comparison under distinctive node mobility.

Since nodes typically exhibit a certain degree of mobility, their clocks may drift if they are inconsistent because of the changes in water. Therefore, MSE fluctuations may occur.  Figure 7 shows MSE comparison under distinctive node mobility. It is worth mentioning that the MSE of the four algorithms fluctuates. In general, the MSE of MU-Sync is about 0.013 s, that of MM-Sync is 0.011 s, that of DE-Sync is 0.009 s, and that of PCDE-Sync is 0.005 s.

For MU-Sync, it considers the relative movement in the two-stage operation, namely the skew and offset acquisition stage and the synchronization stage. For MM-Sync, it establishes a mobile model to analyze the location change and propagation delay, and then, it solves the synchronization issue using one-way communication. For DE-Sync, it substitutes the Doppler scale factor into the linear regression directly to estimate the clock drift and offset. However, partial clustering is achieved in PCDE-Sync, and the stability of clusters is reached. The clock correction and compensation are performed by the Doppler effect in a small region. Consequently, the synchronization accuracy is higher.

### 5.4. The Comparison of Energy Consumption

**Figure 8:**
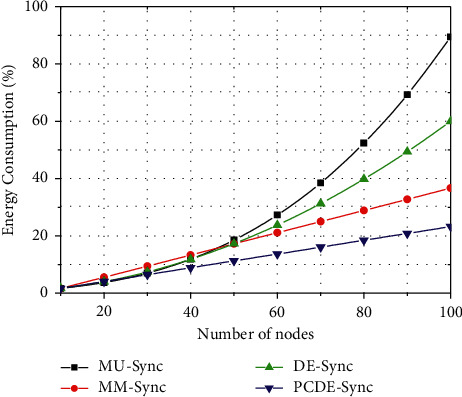
Comparison of energy consumption.

Energy consumption is an essential issue to be considered in UANs. Some synchronization algorithms rely on high-performance hardware, which will increase the development cost and quickly exhaust the energy. Energy consumption mainly depends on complexity.  Figure 8 shows the energy consumption of the four algorithms. When the number of nodes is less than 30, the energy consumption of the four algorithms tends to be the same. However, when nodes increase, the energy consumption will also increase. Among them, those of MU-Sync and DE-Sync change exponentially. When the number is 100, the energy consumption of MU-Sync is as high as 45.3%, that of DE-Sync is 39.7%, that of MM-Sync is 28.6%, and that of PCDE-Sync is 22.9%.

For MU-Sync, it performs linear regression to estimate clock deviation and deploys a gradient descent algorithm to solve multifeature regression with a complexity of *O*(*n*^3^). For DE-Sync, it substitutes the Doppler scale factor to estimate the clock drift and the offset. Therefore, the complexity is *O*(*mn*^2^), where *m* is the number of iterations. For MM-Sync, a one-way communication method is adopted, and there is no need to respond to the synchronization request. Thus, the complexity is *O*(*n*^2^). For PCDE-Sync, it uses AFSA to form partial clustering. Since CH is the time base of a cluster, the electing of CH is limited to the initial cluster, which narrows the communication range. The communication overhead is reduced through intracluster synchronization and intercluster synchronization. Therefore, the complexity is *O*(*kn*), where *K* is the number of clusters. In summary, the complexity of PCDE-Sync is the lowest.

## 6. Conclusions

This paper introduces PCDE-Sync, a novel time synchronization algorithm for underwater acoustic networks. Firstly, we propose a partial clustering method and model the CHs electing process based on the preying, swarming, and following behavior of the fish. Secondly, we design the synchronization procedure and realize the clock correction and compensation founded on the Doppler effect. Finally, we compare the performance of PCDE-Sync with that of MU-Sync, MM-Sync, and DE-Sync in terms of the cumulative error after synchronization, the mean square error under different clock skew and that under distinctive node mobility, and the energy consumption. Simulation results show that PCDE-Sync achieves a trade-off of accuracy and energy consumption when solving synchronization issues. In conclusion, PCDE-Sync provides desirable performance for UANs.

Currently, we are prototyping the underwater nodes of PCDE-Sync to build a lightweight UAN system. We have designed a UAN based on the software-defined networking (SDN) architecture [58–61]. It is worth mentioning that the development process of UAN can be significantly reduced, and the experimental construction can be easily realized based on SDN. Next, field deployment and small-scale experiments will be the main work.

## Figures and Tables

**Algorithm 1:**
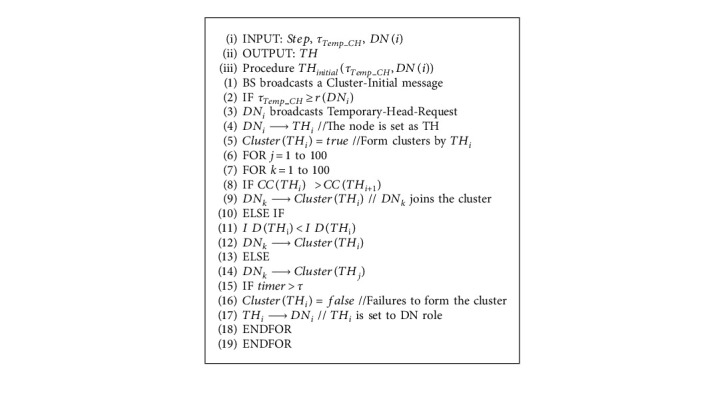
Algorithm 1 Cluster initialization

**Algorithm 2:**
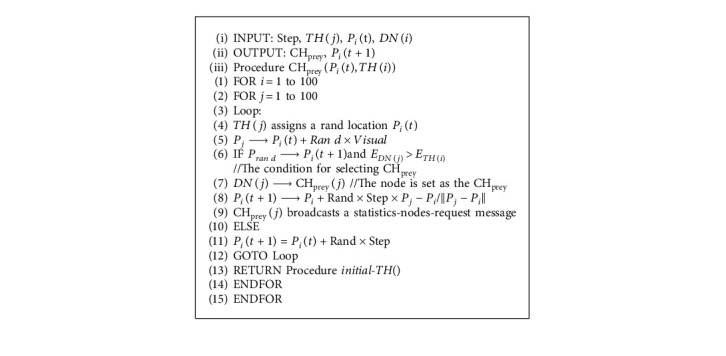
Algorithm 2 CH selection based on the preying behavior

**Algorithm 3:**
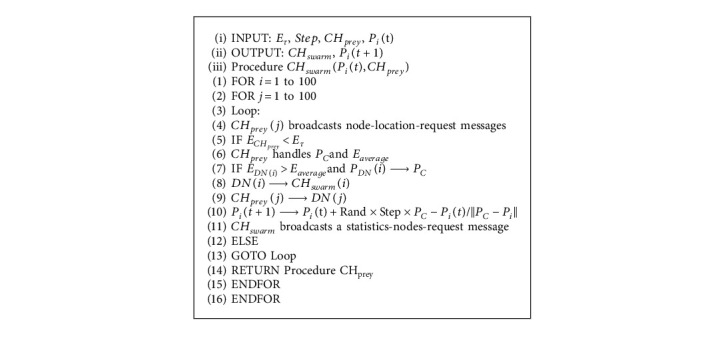
Algorithm 3 CH selection based on swarming behavior

**Algorithm 4:**
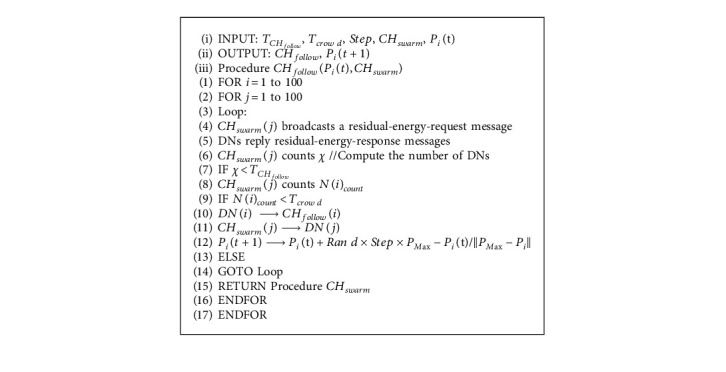
Algorithm 4 CH selection based on following behavior

**Table 1:** The messages are defined for the clustering and synchronization algorithm.

Message name	Sender	Receiver	Description
Cluster-initial	BS	DN	To trigger the partial clustering
Compete–CH–request	TH	DN	TH requests to compete for the CH
Compete–CH–response	DN	TH	DNs respond to the request of TH
Statistics-nodes-request	CH	DN	The CH requests to statistic DNs
Statistics-node-response	DN	CH	DNs response statistic requests
Node-location-request	CH	DN	CH requests the location of DNs
Node-location-response	DN	CH	DNs respond to the location
Residual-energy-request	CH	DN	CH requests the energy of DNs
Residual-energy-response	DN	CH	DNs respond to the residual energy
Cluster-statistics	CH	BS	The CH send the cluster-statistics
Inter-sync-trig	BS	CH	To trigger the synchronization
Inter-sync-req	CH	BS	CHs request synchronization
Inter-sync-ack	BS	CH	BS acknowledges synchronization
Inter-sync-req	DN	CH	The intracluster synchronization
Inter-sync-ack	CH	DN	DNs acknowledge synchronization

**Table 2:** Simulation parameters.

Parameter	Value	Parameter	Value
System clock	8 MHz	Time clock	32.768 KHz
Channel type	Rayleigh	Noise type	Gaussian
Clock offset	800 ms	Clock skew	10–80 PPM
SNR	15 dB	Carrier frequency	30 KHz
Communication range	1000 m	Depth of node	10–100 m
Doppler frequency shift	20 Hz	Doppler spread factor	0.3–0.8
Acoustic velocity	1430–1500 m/s	Node mobility	1–10 m/s
Cyclic prefix	51.2 ms	Multipath delay	40 ms
Threshold of energy	0.35	Threshold of BER	0.005

## Data Availability

The data used to support this study are available from the corresponding author upon request.

## Abbreviations

AFSA: Artificial fish swarm algorithm
APE-Sync: Adaptive synchronization
BS: Base station
CC: Communication capability
CD-Sync: Clustering- and doppler velocity-based synchronization
CH: Cluster head
CLUSS: Cluster-based Secure Synchronization
C-Sync: Clustering-based synchronization
C-TPSN: Chained timing-sync for sensor network
DCH-Sync: Mobile beacon and dual CHs synchronization
DEKCS: Distance and energy-constrained K-means clustering scheme
DE-Sync: Doppler-enhanced synchronization
DN: Data node
D-Sync: Doppler-shift-based synchronization
FITS: Forwarding-based intercluster synchronization
FrFT: Fractional fourier transform
HFM: Hyperbolic frequency modulation
K-Sync: Kalman-filter-based synchronization
LEACH: Low-energy adaptive clustering hierarchy
LFM: Linear frequency modulation
MM-Sync: Motion-based synchronization
MU-Sync: Cluster-based synchronization for mobile UAN
OFDM: Orthogonal frequency division multiplexing
OITS: One-way intracluster time synchronization
PPM: Parts per million
RMS: Root mean square
RMSE: Root means square errors
ROS: Receiver-only synchronization
TH: Temporary head
TITS: Threshold-based intracluster synchronization
TPSN: Timing-sync for sensor network
TSHL: Time synchronization for high latency
UANs: Underwater acoustic networks.
